# Acute and chronic systemic inflammation associated with canine nodular splenic lesions composed of heterogeneous cell components: four cases (2020‐2024)

**DOI:** 10.1111/jsap.13826

**Published:** 2025-01-15

**Authors:** K. Mourou, Y. Abou Monsef, S. Belluco, M. Penent, M. Delverdier, M. Hugonnard, F. Granat, R. Lavoue, M. Mantelli

**Affiliations:** ^1^ Service de Médecine Interne, Département des Animaux de Compagnie de Loisir et de Sport Université de Lyon, VetAgro Sup, Campus Vétérinaire de Lyon Marcy‐l'Étoile France; ^2^ Laboratoire d'Histopathologie Vétérinaire, Université de Toulouse, École Nationale Vétérinaire de Toulouse Toulouse Cedex France; ^3^ Laboratoire d'Histopathologie Vétérinaire, VetAgro Sup, Campus Vétérinaire Marcy l'étoile France; ^4^ Laboratoire Central de Biologie Médicale, ENVT Toulouse France; ^5^ METAML, CRCT, Inserm, ENVT Toulouse France; ^6^ Université de Toulouse Toulouse France; ^7^ INTHERES, Université de Toulouse, INRA, ENVT, UPS Toulouse France

## Abstract

**Objectives:**

To describe the clinical presentation and clinicopathological findings of dogs with nodular splenic lesions composed of heterogeneous cell components associated with systemic inflammation and to provide information on the outcome after surgical resection.

**Materials and Methods:**

Medical records were searched for dogs with histologically and immunohistochemically characterised nodular splenic lesions with mixed stromal, histiocytic and lymphoid cells and the presence of systemic inflammatory markers at the time of diagnosis.

**Results:**

Four dogs were included, of which three had an undifferentiated splenic stromal sarcoma and one had a splenic leiomyosarcoma. Fever and abdominal pain were reported in three and four cases, respectively. All dogs showed hyperglobulinaemia and marked changes in the serum protein electrophoresis profile. C‐reactive protein and fibrinogen concentrations were both increased in three cases. These abnormalities completely resolved after splenectomy. Moreover, two dogs had concomitant glomerular disease and one dog had liver amyloidosis. Three dogs were still alive and asymptomatic 1, 6 and 9 months after surgery. One dog died 16 months after the initial presentation due to complications related to progressive renal failure.

**Clinical Significance:**

Based on this report, nodular splenic lesions with heterogeneous cell components may directly be associated with a pro‐inflammatory state and should be considered as part of the differential diagnosis of fever and hyperglobulinaemia in dogs. Furthermore, early recognition and treatment of these lesions could reduce the risk of systemic complications potentially associated with amyloid deposit and organ failure.

## INTRODUCTION

Splenic masses are a common and potentially life‐threatening condition in middle‐aged to older dogs. Nodular splenic lesions represent a heterogeneous group comprising several pathological entities with different biological behaviour. Forty‐eight to 59% of these lesions are malignant (Backschat et al., 2012; Figueiredo et al., 2019; O'Byrne & Hosgood, 2019; Sherwood et al., 2016; Van den Broek Campanelli et al., 2021). Recent studies have identified hemangiosarcoma (HSA) as the most common splenic malignant neoplasm and lymphoid nodular hyperplasia (LNH) as the most common benign nodular lesion diagnosed among splenectomised dogs (Eberle et al., 2012; Fernandez et al., 2019; Spröhnle‐Barrera et al., 2022).

In the past, a significant number of nodular splenic lesions composed of a mixed population of histiocytoid, spindle and lymphoid cells have fallen under the umbrella term of splenic fibrohistiocytic nodule (SFHN) (Spangler & Kass, 1998). Recently, the use of immunohistochemistry (IHC) allowed proper identification of several specific diseases, including among others LNH, complex nodular hyperplasia (CNH), indolent lymphoma, splenic stromal sarcoma (SSS) and histiocytic sarcoma (HS) (Ferrari et al., 2024; Ko et al., 2013; Moore et al., 2012; Morris et al., 2002; Wittenberns et al., 2021). However, the classification of canine splenic nodules with heterogeneous cell components remains challenging, as a significant number of lesions cannot be attributed to one specific origin due to histomorphological overlap. In particular, a continuum may exist between benign CNH, sarcoma arising from CNH and malignant undifferentiated SSS (Wittenberns et al., 2021). In a recent study, histological criteria of malignancy and distant metastases were reported in several cases of splenic nodules with mixed components for which IHC did not allow for further subclassification. These lesions were designated as “complex splenic tumor,” and the term CNH was used for the remainder (Sabattini et al., 2022).

Although nodular splenic lesions with mixed components are frequently diagnosed in dogs, little information is available about their clinical and clinicopathological features. Reported clinical signs are mostly nonspecific and include weakness, decreased appetite, vomiting and weight loss (Ferrari et al., 2024; Lee et al., 2018; O'Brien et al., 2013; Sabattini et al., 2018). Biological alterations such as anaemia, neutrophilia, platelet disorders, increased liver enzymes and decreased albumin/globulin (A/G) ratio may occur frequently (Ferrari et al., 2024; Latifi et al., 2020; O'Brien et al., 2013; Sabattini et al., 2018; Sabattini et al., 2022; Spangler & Kass, 1998), but haematological and biochemical changes after surgery have yet to be reported.

In our hospitals, we have seen several cases of splenic nodules composed of heterogeneous cell components associated with both acute and chronic inflammatory states. In these cases, markers of systemic inflammation normalised after removal of the nodules by splenectomy. A search of the Medline (Pubmed) database was performed in March 2024 and November 2024 using the following keywords: “splenic mass,” “splenectomy,” “nodular splenic lesion,” “fibrohistiocytic nodule,” “lymphoid nodular hyperplasia,” “complex nodular hyperplasia,” “splenic stromal sarcoma,” “undifferentiated splenic stromal sarcoma” and “complex splenic tumor,” in conjunction with the search terms “dog” and “canine.” In addition, textbooks including Ettinger et al. (2024), Vail et al. (2019), Maxie (2015), and Raskin et al. (2022) were consulted as references. Based on the literature search, this is the first case series to report the clinicopathological follow‐up of dogs with nodular splenic lesions composed of heterogeneous cell components after splenectomy and to describe a direct association between these lesions and severe systemic inflammation.

## MATERIALS AND METHODS

### Study design and inclusion criteria

The medical records of two veterinary teaching hospitals were retrospectively reviewed for dogs diagnosed with splenic nodular lesions composed of a mixed population of histiocytoid, spindle, and lymphoid cells between January 2020 and February 2024. Dogs were included based on the presence of systemic inflammatory indicators at the time of diagnosis. Cases were excluded if a thorough characterisation of the splenic nodule by histology and IHC was not performed.

### Medical record search and data extraction

One investigator searched the clinical database software using the following keywords: “splenic mass,” “splenectomy,” “nodular splenic lesion,” “fibrohistiocytic nodule,” “lymphoid nodular hyperplasia,” “complex nodular hyperplasia,” “splenic stromal sarcoma,” “undifferentiated splenic stromal sarcoma” and “complex splenic tumor.” The species was limited to “dog.” For all dogs eligible for inclusion, data collected from the medical records included signalment, clinical signs, physical examination findings, results of clinicopathological tests, histological diagnosis, treatment, complications and outcome.

## RESULTS

Seventy‐six dogs underwent splenectomy for resection of a splenic mass during the study period. Of these, 18 dogs (24%) had a splenic nodular lesion composed of mixed cell components. Ten were classified as LNH, four as undifferentiated SSS, three as CNH and one as splenic leiomyosarcoma. Four dogs met the inclusion criteria, including three dogs with undifferentiated SSS and one dog with splenic leiomyosarcoma. Fever, abdominal pain, marked changes in the serum protein electrophoresis (SPE) profile, increased C‐reactive protein (CRP) concentration and/or hyperfibrinogenaemia were reported for each at presentation and completely resolved after splenectomy.

### Case 1

An 11‐year‐old neutered male mixed breed dog, weighing 27 kg (BCS, body condition score, of 1/9), was presented with acute onset of anorexia, vomiting, diarrhoea and a distended, painful abdomen. Chronic vomiting and progressive weight loss were also reported. On physical examination, the dog presented severe cachexia and moderate hyperthermia (39.4°C). Abdominal palpation was painful, and a large mass was identified.

**FIG 1:**
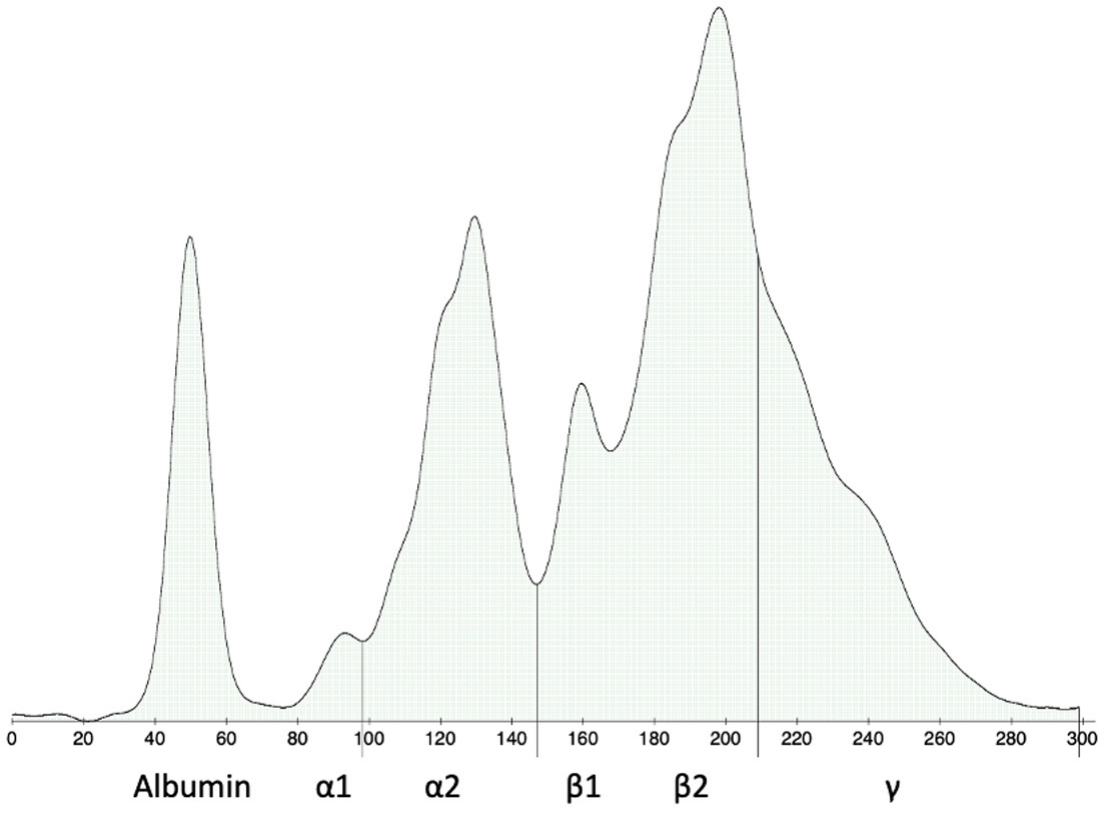
Serum protein electrophoresis. Typical electrophoretic changes from case 1, including broad peaks in α2 and β2 regions, polyclonal hypergammaglobulinaemia, beta‐gamma bridging and marked hypoalbuminaemia.

**FIG 2:**
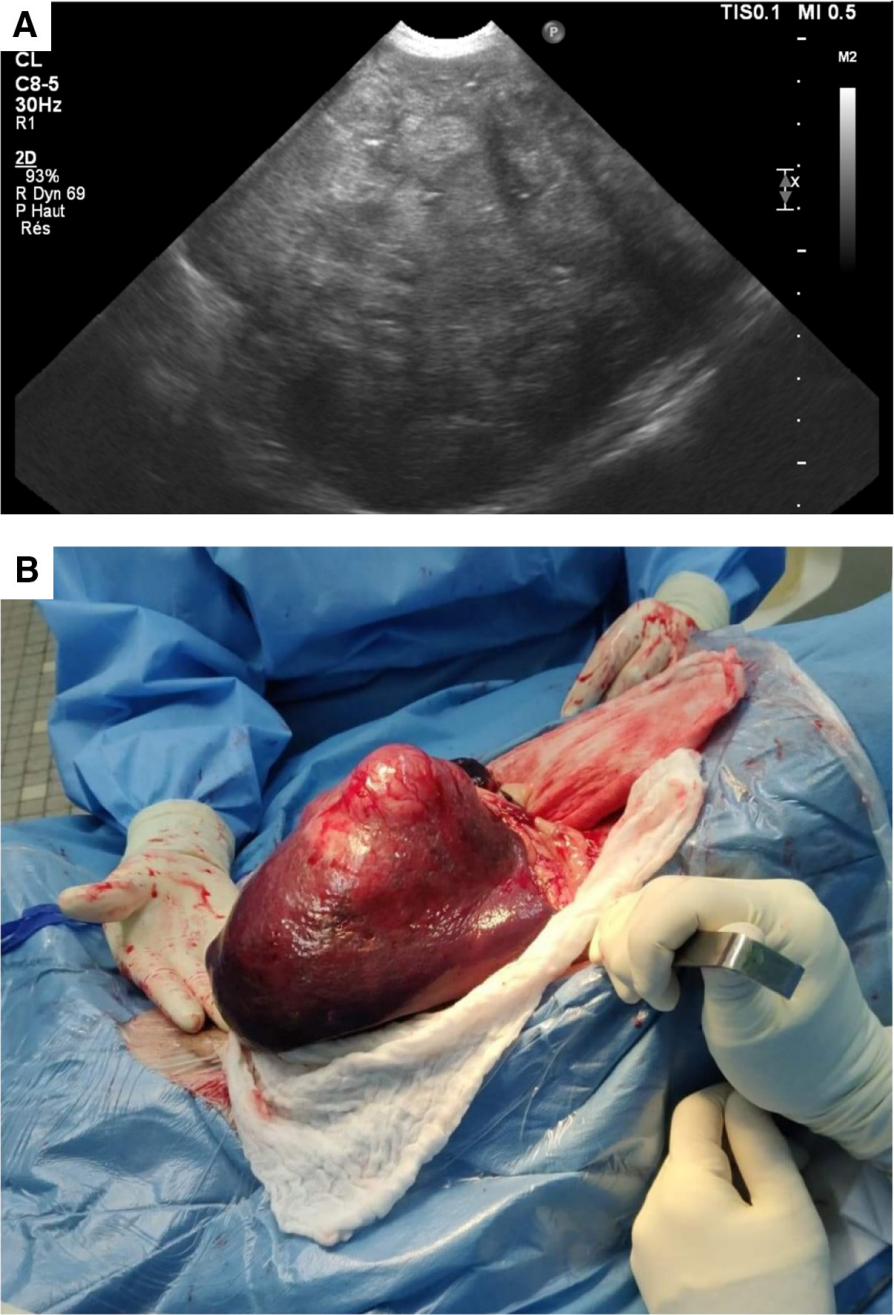
Ultrasonographic (A) and macroscopic (B) aspect of a nodular splenic lesion.

**FIG 3:**
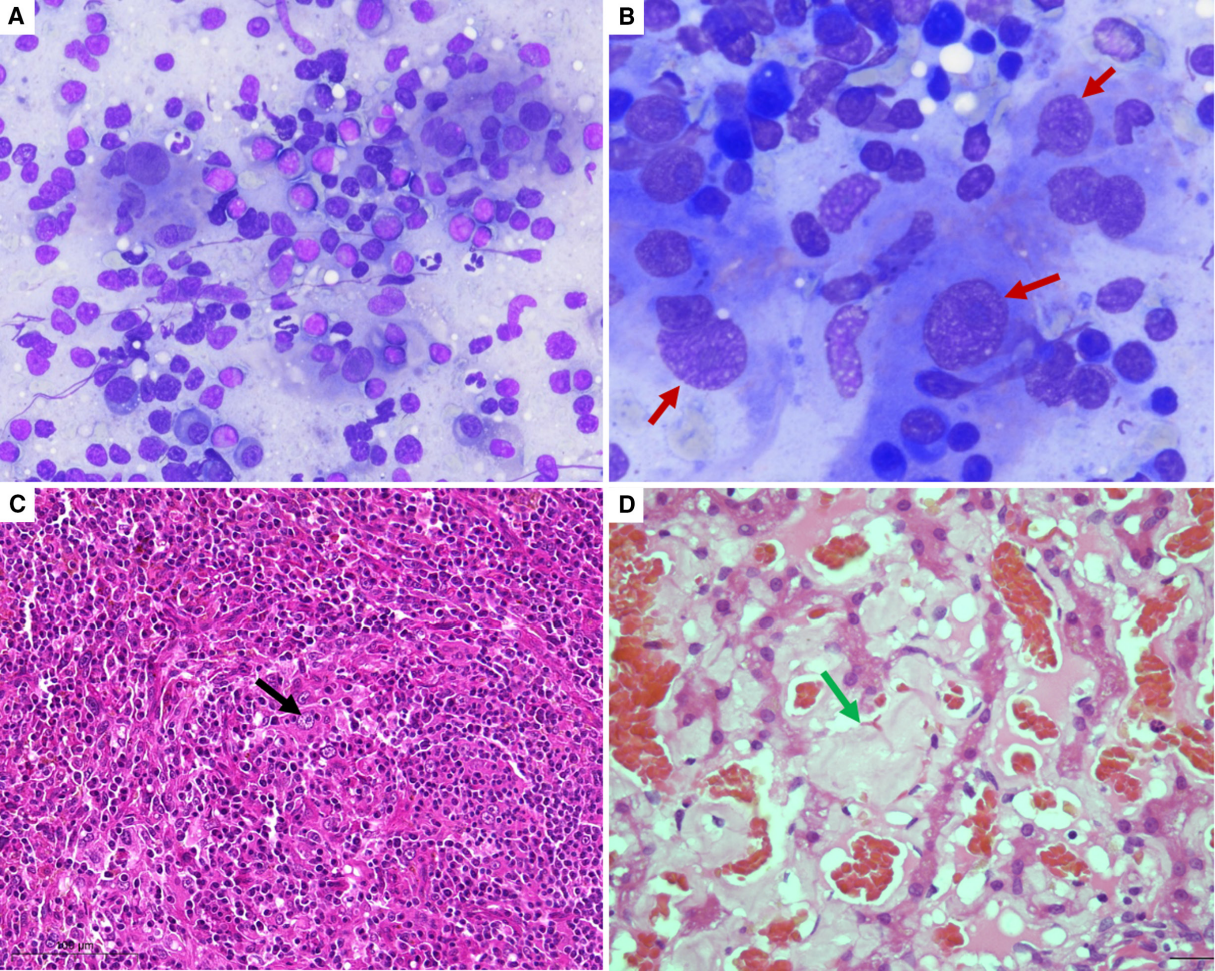
Cytological (A, B) and histopathological (C, D) features of case 1. (A) Spleen, May‐Grünwald‐Giemsa (MGG) staining, ×200: mixed population with a majority of lymphoid cells, some neutrophils and few atypical spindle cells. (B) Spleen, MGG staining, ×400: atypical spindle cells (red arrows) showing some atypia (anisokaryosis, anisocytosis, nucleolation and binucleation) admixed with diffuse lymphoplasmacytic cells. (C) Spleen, haematoxylin and eosin (HE): mixed proliferation of lymphocytes, histiocytes and stromal cells with prominent nuclear atypia (black arrow). (D) Liver, HE: amyloid deposits in the subendothelial spaces (green arrow) leading to pressure atrophy of the hepatocytes.

Complete blood cell count (CBC) showed a mild, microcytic, hypochromic, non‐regenerative anaemia and a mild lymphopenia. Biochemical abnormalities included increased creatinine concentration, marked hyperglobulinaemia, mild hyperfibrinogenaemia, increased alkaline phosphatase (ALKP) activity, and mild hypercholesterolaemia. Resting and postprandial serum bile acid concentrations were consistent with hepatobiliary dysfunction (Table 1). SPE revealed a severe hypoalbuminaemia and a marked hyperglobulinaemia with broad peaks in the α2 and β2 regions, polyclonal hypergammaglobulinaemia and beta‐gamma bridging (Fig 1). Urinalysis showed marked proteinuria with a urinary protein‐to‐creatinine (UPC) ratio of 13.3 (RI, <0.5). Urine protein electrophoresis was consistent with mixed, non‐selective (i.e., both glomerular and tubular) proteinuria. Abdominal ultrasound showed a 20 cm splenic mass with heterogeneous appearance and irregular but encapsulated borders, and abnormal liver appearance characterised by heterogeneous parenchyma and target‐like lesions (Fig 2A). Thoracic radiography revealed a mild broncho‐interstitial pattern in the overall lung field. Echocardiography was unremarkable. Cytological examination of the spleen revealed severe plasmacytic hyperplasia associated with granulomatous infiltration and the presence of some atypical spindle cells of uncertain origin (Fig 3A, B). Fine needle aspirate of the liver was consistent with marked cholestasis and inflammation, associated with large amounts of amorphous pink extracellular matrix between hepatocytes. Based on these findings, amyloidosis was suspected.

**Table 1:** Results of CBC, biochemistry profile, electrolyte panel and coagulation tests obtained on the day of admission for the four cases

Variables (unit)	Case 1	Case 2	Case 3	RIs	Case 4	RIs
Haematology						
PCV (%)	34.5	37.0	18.9	37.0 to 55.0	32.8	35.0 to 52.0
RBC (10^12^/L)	6.65	7.18	3.36	5.20 to 7.90	4.80	5.10 to 7.90
Haemoglobin (g/dL)	11.7	12.1	5.90	12.4 to 19.2	11.3	12.4 to 19.1
MCV (fL)	51.9	53.5	56.3	60.0 to 71.0	68.0	60.0 to 71.0
MCHC (g/dL)	33.9	31.5	31.2	34.4 to 38.1	34.5	34.0 to 38.0
Reticulocytes (10^9^/L)	36.6	20.8	81.3	19.4 to 150.0	38.6	0.0 to 80.0
WBC (10^9^/L)	8.44	8.75	13.66	5.6 to 20.4	19.0	5.6 to 20.4
Neutrophils (10^9^/L)	7.76	6.39	11.63	2.9 to 13.6	15.6	2.9 to 13.6
Eosinophils (10^9^/L)	0.00	0.00	0.05	0.1 to 3.1	0.20	0.0 to 1.4
Lymphocytes (10^9^/L)	0.16	2.01	1.21	1.1 to 5.3	2.10	1.1 to 5.3
Monocytes (10^9^/L)	0.42	0.26	0.77	0.4 to 1.6	1.00	0.3 to 1.6
Platelets (10^9^/L)	391	390	900	108 to 562	164	140 to 600
Biochemistry						
Glucose (mmol/L)	6.1	5.3	5.4	3.7 to 8.2	4.9	4.1 to 8.8
Creatinine (μmol/L)	241	112	52	44 to 133	105	71 to 212
Urea (mmol/L)	10.3	3.8	4.0	1.6 to 10.9	8.3	5.7 to 12.9
Total protein (g/L)	93	95	62	48 to 66	>120	50 to 69
Albumin (g/L)	25	26	21	23 to 39	35	23 to 34
Globulin (g/L)	67	69	41	19 to 37	>85	24 to 39
A/G ratio	0.4	0.4	0.5	0.8 to 1.3	<0.4	0.8 to 1.3
ALKP (U/L)	791	1396	245	20 to 155	80	14 to 111
ALT (U/L)	11	159	31	3 to 50	286	12 to 130
AST (U/L)	20	67	30	1 to 37	–	–
GGT (U/L)	5	<10	–	5 to 25	–	0 to 2
Total bilirubin (μmol/L)	3.8	3.2	1.7	1.7 to 12.0	59	0 to 15.0
CK (U/L)	46	131	–	25 to 467	–	0 to 310
Cholesterol (mmol/L)	8.6	6.1	–	3.3 to 8.3	–	3.1 to 7.1
Triglyceride (mmol/L)	1.4	1.1	–	0.2 to 1.3	–	0.1 to 1.6
CRP (mg/L)	–	151	230	<10	85	<10
Pre‐prandial bile acids (μmol/L)	33.8	–	–	<20	–	<20
Post‐prandial bile acids (μmol/L)	17.1	–	–	<20	–	<20
Electrolyte panel						
Sodium (mmol/L)	144	144	–	138 to 148	147	144 to 160
Potassium (mmol/L)	4.2	4.2	–	3.2 to 5.0	3.0	3.5 to 5.8
Chloride (mmol/L)	118	115	–	100 to 118	112	109 to 122
Total CO_2_ (mmol/L)	13	21	–	16 to 25	–	–
Total calcium (mmol/L)	3.0	2.7	–	2.4 to 3.0	–	2.2 to 2.8
Ionised calcium (mmol/L)	1.3	1.4	–	1.2 to 1.5	–	1.1 to 1.3
Magnesium (mmol/L)	1.0	0.8	–	0.7 to 1.0	–	0.7 to 1.0
Phosphorus (mmol/L)	1.98	1.20	–	0.7 to 2.6	–	0.8 to 2.3
Coagulation panel						
Fibrinogen (g/L)	7.2	14.1	10.4	1.3 to 4.7	3.9	1 to 5
APTT (seconds)	12.7	16.4	16.1	12.9 to 16.9	14.3	9.7 to 15
PT (seconds)	8.2	9.5	9.2	7.3 to 9.9	8.3	5.9 to 8.4
Antithrombin III activity (%)	95	–	–	102 to 191	–	–

PCV Packed cell volume, RBC Red blood cell, MCV Mean corpuscular volume, MCHC Mean corpuscular haemoglobin concentration, WBC White blood cell, A/G Albumin/globulin ratio, ALKP Alkaline phosphatase activity, ALT Alanine aminotransferase activity, AST Aspartate aminotransferase activity, GGT Gamma‐glutamyl transferase activity, CK Creatine kinase activity, CRP C‐reactive protein concentration, APTT Activated partial thromboplastin time, PT Prothrombin time, RIs Reference intervals

Reference intervals are provided by the laboratory

The colour shadings indicate values below the reference interval are shaded green, while values above the reference interval are shaded red. The more intense the colour in a cell, the higher the value

Splenectomy was performed (Fig 2B) and liver biopsies were obtained. As the dog was unstable under anaesthesia, neither kidney biopsies nor bone marrow aspiration was performed. On histopathology, the resected splenic nodule was poorly demarcated and composed of a pleocellular infiltrate of stromal, histiocytic, plasmacytic and lymphoid components. Spindle cells harboured marked nuclear atypia. Karyomegaly and multinucleated cells were frequent (Fig 3C). The mean mitotic count in 2.37 mm^2^ was 1. Multifocal to coalescing necrosis and splenic vein thrombosis (SVT) were also present. Tumour cells were strongly positive for CD204, Iba1 and desmin, and negative for CD31 and smooth‐muscle actin (SMA). These findings were consistent with an undifferentiated SSS. The non‐nodular splenic regions were of normal architecture with a normal white and red pulp structures. The splenic parenchyma showed mild extramedullary haematopoiesis and venous congestion. Liver histopathology performed with routine and special stains (Masson trichrome and Congo red) revealed amyloidosis and fibrosis (Fig 3D).

Samples of the spleen were submitted for detection of *Anaplasma* spp., *Babesia* spp., *Bartonella* spp., *Ehrlichia* spp. and *Leishmania* spp. by polymerase chain reaction (PCR) assays. Serum samples were tested for the presence of *Anaplasma* spp., *Borrelia burgdorferi* and *Ehrlichia* spp. antibodies and *Dirofilaria immitis* antigen using a qualitative dot‐ELISA SNAP 4Dx Plus test (IDEXX Laboratories; Westbrook, Maine, USA). Quantitative serology testing was performed for both *Bartonella* spp. and *Leishmania infantum*. All the analyses were negative, except for serological testing positivity for *Bartonella henselae* (positive serologic testing 1/100).

The dog recovered uneventfully. He was discharged 3 days later with renal diet, benazepril (0.5 mg/kg PO, per os, q24h) and aspirin (1 mg/kg PO q24h). At discharge, haematological and biochemical results remained stable except for significant improvement of hyperglobulinaemia (Table 2). One month postoperatively, the dog's general condition had improved, and laboratory findings revealed persistent mild hyperglobulinaemia and stable creatinine concentration (Table 2). UPC ratio remained markedly elevated (10.5; RI, <0.5). Benazepril dosage was increased (0.5 mg/kg PO q12h). Six months postoperatively, the dog's condition had markedly improved. Albumin and globulin concentrations were within RIs. Azotaemia worsened mildly; hypercholesterolaemia and increased ALKP activity persisted (Table 2). Urinalysis displayed persistent proteinuria with a significant decrease in UPC ratio (4.6; RI, <0.5). The dog was lost to follow‐up. He was presented 1 year later with marked alteration of general condition and progression of renal disease. Due to poor prognosis, the dog was humanely euthanised 16 months after initial presentation.

**Table 2:** Results of long‐term haematological and biochemical monitoring of case 1

Variables (unit)	Day 1	Day 3	Day 30	Day 180	Day 200	RIs
Haematology						
PCV (%)	34.5	29.7	44.3	43.7	–	37.0 to 55.0
RBC (10^12^/L)	6.65	5.72	8.16	6.86	–	5.20 to 7.90
Haemoglobin (g/dL)	11.7	10.0	15.1	16.0	–	12.4 to 19.2
MCV (fL)	51.9	51.9	54.3	63.7	–	60.0 to 71.0
MCHC (g/dL)	33.9	33.7	34.1	36.6	–	34.4 to 38.1
Reticulocytes (10^9^/L)	36.6	38.3	22.8	55.6	–	19.4 to 150.0
WBC (10^9^/L)	8.44	13.55	7.35	9.15	–	5.6 to 20.4
Neutrophils (10^9^/L)	7.76	11.79	4.99	5.67	–	2.9 to 13.6
Eosinophils (10^9^/L)	0.00	0.00	0.15	0.64	–	0.1 to 3.1
Lymphocytes (10^9^/L)	0.16	0.81	0.96	2.38	–	1.1 to 5.3
Monocytes (10^9^/L)	0.42	0.41	1.10	0.46	–	0.4 to 1.6
Platelets (10^9^/L)	391	289	581	550	–	108 to 562
Biochemistry						
Creatinine (μmol/L)	241	195	224	283	378	44 to 133
Urea (mmol/L)	10.3	–	–	17.8	37.2	1.6 to 10.9
Total protein (g/L)	93	76	75	67	70	48 to 66
Albumin (g/L)	25	24	26	32	34	23 to 39
Globulin (g/L)	67	52	49	35	36	19 to 37
A/G ratio	0.4	0.5	0.5	0.9	0.9	0.8 to 1.3
ALKP (UI/L)	791	–	–	593	–	20 to 155
ALT (UI/L)	11	–	–	55	–	3 to 50
AST (UI/L)	20	–	–	41	–	1 to 37
GGT (UI/L)	5	–	–	8	–	5 to 25
Total bilirubin (μmol/L)	3.8	–	–	2.4	–	1.7 to 12.0
Cholesterol (mmol/L)	8.6	9.4	–	10.9	–	3.3 to 8.3

PCV Packed cell volume, RBC Red blood cell, MCV Mean corpuscular volume, MCHC Mean corpuscular haemoglobin concentration, WBC White blood cell, A/G Albumin/globulin ratio, ALKP Alkaline phosphatase activity, ALT Alanine aminotransferase activity, AST Aspartate aminotransferase activity, GGT Gamma‐glutamyl transferase activity, RIs Reference intervals

The colour shadings indicate values below the reference interval are shaded green, while values above the reference interval are shaded red. The more intense the colour in a cell, the higher the value

### Case 2

A 5‐year‐old neutered male American Bully dog, weighing 37.6 kg (BCS of 4/9), was presented with acute onset of lethargy, anorexia, vomiting and diarrhoea. Prior to presentation, the dog had a 15‐day history of recurrent fever. Physical examination revealed lethargy and pain on abdominal palpation. Rectal temperature was normal (38.7°C).

Laboratory findings included mild microcytic hypochromic non‐regenerative anaemia, marked hyperglobulinaemia, decreased A/G ratio, increased serum concentration of CRP, severe hyperfibrinogenaemia, and increase in ALKP, alanine aminotransferase (ALT) and aspartate aminotransferase (AST) activities (Table 1). SPE revealed a severe hypoalbuminaemia and a marked hyperglobulinaemia with broad peaks in the α2 and β2 regions, polyclonal hypergammaglobulinaemia and beta‐gamma bridging. On urinalysis, the UPC ratio was moderately increased (1.9; RI, <0.5). Abdominal ultrasonography revealed a large splenic mass measuring 130 × 80 mm, located on the dorsal aspect of the spleen, and described as cavitated and heterogeneous. Three‐view thoracic radiography and echocardiography did not reveal any abnormalities.

**FIG 4:**
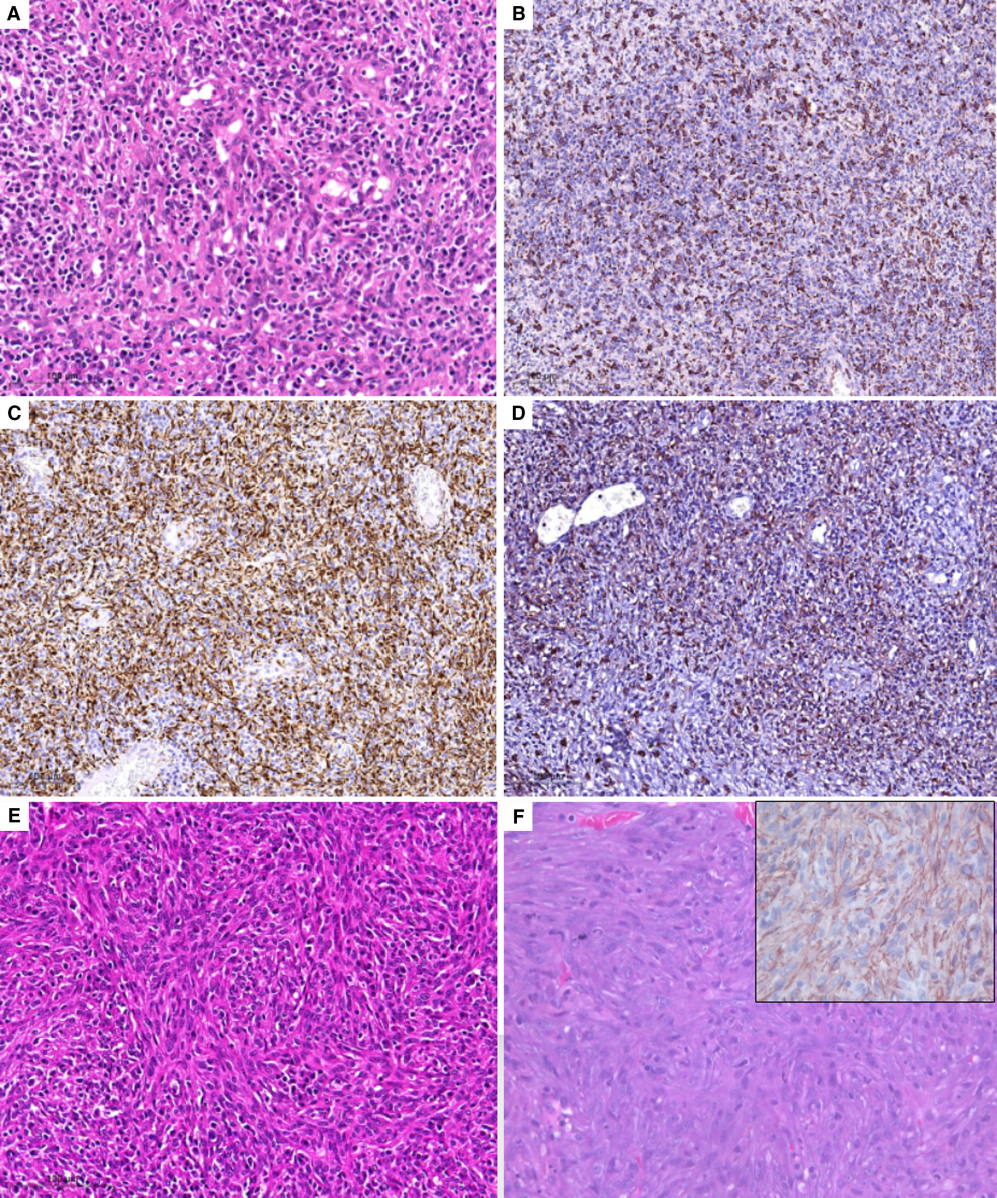
Spleen histopathology (HE) and immunohistochemistry of cases 2, 3 and 4. (A) sparsely distributed lymphoid elements throughout predominantly stromal and histiocytic cell components, with strong IHC positivity of tumor cells for (B) IBA1, (C) desmin, and (D) CD204 in case 2. (E) streaming bundles of neoplastic stromal cells admixed with diffuse lymphoplasmacytic and histiocytic cells in case 3. (F) neoplastic spindle cells forming bundles and staining positive for SMA (inset) in case 4.

Splenectomy was performed. As the anaesthesia was uneventful, bone marrow aspiration was also performed at the end of the procedure for cytologic assessment. Liver biopsies were not available for this dog. Bone marrow fine needle aspirate showed an increased cellularity with a normal myeloid to erythroid (M:E) ratio (0.9; RI, 0.75 to 2.0). Maturation and morphology of erythroid, myeloid and megakaryocytic lineages were normal. Iron stores seemed normal (2/4). The percentage of plasma cells (8.4%) was increased but their morphology was normal. Histopathological examination of the resected spleen revealed a poorly circumscribed nodule composed of sheets of spindle cells showing moderate atypia admixed with infiltrates of histiocytes, plasma cells and lymphocytes predominately arranged in follicles. Karyomegaly and multinucleated cells were occasionally seen (Fig 4A). The mean number of mitoses in 2.37 mm^2^ was 6. The nodule harboured large areas of necrosis. SVT was also present. Tumour cells were strongly positive for Iba1 (Fig 4B), desmin (Fig 4C) and CD204 (Fig 4D), weakly positive for SMA, and negative for CD31. These findings were consistent with an undifferentiated SSS. The adjacent splenic parenchyma was slightly compressed by the expanding effect of the nodule and showed mild extramedullary haematopoiesis. The remainder of the spleen was of normal architecture.

Tissue samples from the resected spleen were submitted for *Bartonella* spp. detection by PCR assay. Serology testing was also performed for *Bartonella* spp. Serum samples were tested for the presence of *Anaplasma* spp., *B. burgdorferi* and *Ehrlichia* spp. antibodies and *D. immitis* antigen using a qualitative dot‐ELISA SNAP 4Dx Plus test (IDEXX Laboratories; Westbrook, Maine, USA). Results were all negative.

Recovery from surgery was uneventful. Two weeks postoperatively, the dog's general condition was considered normal by the owner. No fever was reported, and gastrointestinal signs had completely resolved. Haematological results remained stable. Biochemical analysis revealed a significant decrease in total protein (68 g/L; RI, 48 to 66 g/L) and globulin (39 g/L; RI, 19 to 37 g/L) concentrations with a mild increase of A/G ratio (0.73; RI, 0.8 to 1.3). Serum concentration of CRP was within RI. Mild increase in ALKP (184 U/L; RI, 20 to 155 U/L) and ALT (65 U/L; RI, 3 to 50 U/L) activities persisted. Analysis of a voided urine sample was unremarkable. A telephonic follow‐up was performed 9 months after discharge and the owner reported no evidence of clinical signs.

### Case 3

An 8‐year‐old spayed female American Staffordshire terrier dog, weighing 14.6 kg (BCS, 4/9), was presented for evaluation of a splenic mass and anaemia. Prior to presentation, the dog had a 3‐week history of progressive lethargy and decreased appetite. Physical examination only revealed discomfort on abdominal palpation. Rectal temperature was normal (38.9°C).

CBC and biochemistry results included microcytic hypochromic mildly regenerative anaemia, thrombocytosis, hypoalbuminaemia and hyperglobulinaemia with decreased A/G ratio, increased serum concentration of CRP, severe hyperfibrinogenaemia, and increased ALKP activity (Table 1). SPE revealed a severe hypoalbuminaemia and a broad peak in α2 region. On urinalysis, the UPC ratio was increased (4.66; RI, <0.5). The urine protein electrophoresis was consistent with glomerular proteinuria. Abdominal ultrasonography revealed a heterogeneous splenic mass measuring 50 × 80 mm, located on the cranial extremity of the spleen, and described as cavitated and heterogeneous. A small amount of peritoneal free fluid was also present. Three‐view thoracic radiography and echocardiography did not reveal any abnormalities.

Splenectomy was performed. As anaesthesia was uneventful, bone marrow aspiration was also performed at the end of the procedure. Liver biopsies were not available for this dog. Bone marrow fine needle aspirate showed an increased cellularity with a normal M:E ratio (1.3; RI, 0.75 to 2.0). Maturation and morphology of erythroid, myeloid and megakaryocytic lineages were normal. The percentage of plasma cells (12%) was increased with some signs of activation. A slight increase in the percentage of macrophages (0.7%) was suspected. Iron stores appeared to be decreased (1/4), but no specific staining was performed to confirm this observation. Histopathological examination of the resected spleen revealed a poorly demarcated mass mainly comprising stromal, histiocytic and lymphoplasmacytic elements. The lymphoplasmacytic population was composed of mature lymphocytes associated with plasma cells predominately arranged in follicles. Spindle cells were arranged in streaming bundles and showed moderate to marked nuclear atypia (Fig 4E). The mean number of mitoses in 2.37 mm^2^ was 5. The nodule harboured a high degree of necrosis. SVT was also present. Tumour cells were strongly positive for CD204, Iba1 and desmin and negative for CD31 and SMA. These findings were consistent with an undifferentiated SSS. The remainder of the spleen was of normal architecture and showed a mild degree of extramedullary haematopoiesis and venous congestion.

Recovery from surgery was uneventful. Two weeks postoperatively, the dog was bright and alert with an excellent appetite. Haematological results revealed an improvement of red blood cell variables. Biochemical analysis revealed persistent hypoalbuminaemia (22 g/L; RI, 23 to 39 g/L) and a decrease of total protein (55 g/L; RI, 48 to 66 g/L) and globulin (33 g/L; RI, 19 to 37 g/L) concentrations to within RIs, with a mild increase of A/G ratio (0.7; RI, 0.8 to 1.3). UPC ratio remained increased (3.72; RI, <0.5). A telephonic follow‐up was performed 1 month after discharge and the owner reported no recurrence of clinical signs.

### Case 4

An 8‐year‐old intact male Airedale Terrier dog, weighing 25.3 kg (BCS, 5/9), was referred with a 3‐day history of weakness, anorexia and pigmenturia. Fever (40.1°C) was also reported by the referring veterinarian. Physical examination revealed lethargy, moderate hyperthermia (39.4°C) and abdominal pain.

Laboratory findings included mild normocytic normochromic non‐regenerative anaemia, mild neutrophilia, marked hyperglobulinaemia, decreased A/G ratio, increased serum CRP concentration, increased ALT activity, marked hyperbilirubinaemia and moderate hypokalaemia (Table 1). SPE revealed a moderate hypoalbuminaemia and a polyclonal hypergammaglobulinaemia. Saline agglutination test and direct Coombs test were negative. UPC ratio was mildly increased (0.6; RI, <0.5). Abdominal ultrasound identified mild peritoneal effusion, hyperechoic mesenteric fat, presence of a large, cavitated, heterogeneous mass located on the body of the spleen and mildly enlarged splenic lymph node. Three‐view thoracic radiography was unremarkable. Spleen cytology was suggestive of lymphoid hyperplasia with neutrophilic infiltration.

Surgical exploration revealed a ruptured splenic mass approximately 8 cm in diameter. The dog underwent splenectomy. Liver biopsies and bone marrow aspiration were declined by the owner. Histopathological examination of the resected spleen revealed the presence of two coalescing different lesions: a LNH, characterised by numerous large lymphoid follicles separated by a red pulp rich in reticular cells and iron‐laden macrophages, and an infiltrating leiomyosarcoma, composed of bundles of tumoral spindle cells, in a scant fibrous matrix. Anisocytosis and anisokaryosis were moderate and five mitoses in 2.37 mm^2^ were present. Tumour cells were negative for CD204, Iba1 and CD31, and strongly positive for desmin and SMA (Fig 4F). The remaining splenic parenchyma was unremarkable.

Recovery from splenectomy was uneventful. Hyperthermia resolved within 24 hours of surgery. At discharge, CBC was unremarkable. ALT activity as well as globulin and bilirubin concentrations were within RIs. Urinalysis was unremarkable. After 1 month, the dog remained bright. Physical examination was unremarkable. Biological monitoring and adjuvant chemotherapy were declined by the owner. A telephonic follow‐up was performed 6 months postoperatively and the owner reported that the dog was still asymptomatic.

## DISCUSSION

Canine nodular splenic lesions with heterogenous cell components represent a complex group of specific diseases with distinct biological behaviour and prognosis, from benign LNH to malignant SSS/HS. To date, data regarding the clinical and clinicopathological features of such lesions remain scarce, regardless of their nature.

In the present study, three of the four dogs had fever reported in the recent medical history. Significant pain on abdominal palpation was recorded in all four dogs at presentation. Moreover, two dogs presented with vomiting and diarrhoea and one dog had a history of chronic weight loss. Information about clinical signs associated with nodular splenic lesions other than HSA have seldom been reported in the literature. In addition, the clinical presentation is comprised of a variety of mostly nonspecific clinical signs. In a recent study describing 37 dogs with nodular splenic lesions composed of mixed components, 66.7% were symptomatic, presenting lethargy, anorexia, vomiting and weight loss (Sabattini et al., 2022). These clinical signs were also commonly reported in previous studies of dogs with splenic lymphoid nodules including LNH, CNH, marginal zone lymphoma and SSS (Ferrari et al., 2024; O'Brien et al., 2013; Sabattini et al., 2018). Although abdominal pain was occasionally described in the aforementioned studies, fever was never reported, in contrast to our findings. Fever in dogs with nodular splenic lesions may be secondary to the lesions themselves or associated with peritonitis, as rapid and persistent resolution was documented following total splenectomy in our case series. These findings suggest that nodular splenic lesions should be included in the differential diagnosis of fever in dogs.

All four dogs in the present case series had hyperglobulinaemia and significantly decreased A/G ratio. SPE showed decreased albumin in all dogs, a broad peak in α2 region and polyclonal hypergammaglobulinaemia in three dogs each, and broad peak in β2 region with beta‐gamma bridging in two dogs, as commonly seen in acute and chronic inflammatory processes (Tappin et al., 2011). Renal protein loss may also have contributed to the hypoalbuminemia in cases 1 and 3. These findings are in accordance with a previous study reporting decreased A/G ratio secondary to polyclonal gammopathy in up to 60% of dogs with nodular splenic lesions with mixed components (Sabattini et al., 2022). However, another recent study reported hyperglobulinaemia in only 14.3% of dogs with SSS (Ferrari et al., 2024). In our series, the presence of a severe systemic inflammation was also supported by hyperfibrinogenaemia in cases 1, 2 and 3, increased CRP in cases 2, 3 and 4, as well as cytologic examination of bone marrow in cases 2 and 3. In the previous reports, the inflammatory status was not followed after splenectomy. The resolution of hyperglobulinaemia and the return of inflammatory markers to normal values support the hypothesis that nodular splenic lesions with mixed components might be directly associated with both acute and chronic inflammatory states in dogs. As already suggested, hyperglobulinaemia might correlate with the histologic finding of inflammatory cell infiltration in nodular splenic lesions, including lymphocytes and plasma cells (Sabattini et al., 2022).

Further relevant clinicopathological findings in the present report included mild to moderate non‐regenerative anaemia and elevated liver enzymes in all four cases. In one previous study of 98 dogs with so‐called SFHN, haematological and biochemical features were not consistent enough to provide useful diagnostic or prognostic information but included evidence of anaemia and elevated serum ALKP activity (Spangler & Kass, 1998). Anaemia has also been reported in other studies of dogs with HSA, HS, SSS, splenic nodular lymphoid lesions and splenic nodules with mixed components (Ferrari et al., 2024; Latifi et al., 2020; Lee et al., 2018; O'Brien et al., 2013; Sabattini et al., 2018; Sabattini et al., 2022). In our series, anaemia of inflammatory disease was the main presumptive diagnosis. However, given the erythroid hyperplasia without increase in iron stores in cases 2 and 3, chronic bleeding induced by the mass was also considered. The microcytic, hypochromic nature of the anaemia in cases 1, 2 and 3 further supported this hypothesis. Chronic kidney disease and hepatic amyloidosis may also have contributed to the non‐regenerative anaemia in case 1. Finally, the marked hyperbilirubinaemia that normalised after surgery in case 4 was consistent with either microangiopathic haemolytic anaemia or resorption of the hemoabdomen or functional cholestasis. However, bone marrow aspiration and bone marrow biopsy would have been ideal in all four dogs to investigate their anaemia. Liver biopsies would have been required in cases 2, 3 and 4 to make a definitive diagnosis and to look for metastatic spread, reactive hepatopathy or complications of nodular splenic lesions. In case 3, recent administration of corticosteroid could also have explained the increase in ALKP activity.

Cytological examination of the spleen may be a safe and valuable tool for differentiating between inflammatory, benign and malignant nodular lesions. This may help to prompt surgery or prevent unnecessary splenectomy (Tecilla et al., 2019). Although there may be concerns about seeding of neoplastic cells along the fine needle aspiration tract, this has never been reported for splenic masses or sarcomas (Klopfleisch et al., 2011). However, a definitive diagnosis can only be made by histopathological examination of the spleen after splenectomy.

SVT was detected on histopathology in three of the four cases and has previously been reported in 37% of dogs with nodular splenic lesions composed of mixed components (Sabattini et al., 2022). In our series, SVT was only microscopic, not reported on ultrasound, and was not associated with serious splenic sequelae such as splenic infarction. Several conditions may have been associated with the aetiopathogenesis of SVT in our cases, including local venous congestion or vascular compression, or hypercoagulable state secondary to underlying neoplasia, systemic inflammation or protein‐losing nephropathy (Laurenson et al., 2010). Hyperfibrinogenaemia may also have caused hypercoagulability (Vilar Saavedra et al., 2011; Wennogle et al., 2021). Therefore, preoperative and postoperative evaluation of coagulation using thromboelastometry/thromboelastography would have been ideal to assess the risk of thrombosis and the relevance of thromboprophylaxis in our cases.

No information is available regarding the influence of the size of these nodular splenic lesions. In the literature, it is reported that neoplastic lesions are significantly larger than non‐neoplastic ones using a 2.5 cm cut‐off value (Corvera et al., 2024; Lee et al., 2018). In our study, all masses were larger than 2.5 cm. Given the marked inflammation associated with these lesions, it is possible that the largest nodules are associated with the most severe inflammation and clinical signs. Case 1 was presented with the most chronic history and the largest lesion of the four cases. The dog also displayed the most severe clinical presentation. Thus, increased awareness of nodular splenic lesions is indicated as a prompt detection and management could allow for a better recovery and prognosis without long‐term irreversible complications.

Hepatic amyloidosis was diagnosed in case 1. In veterinary medicine, reactive amyloidosis is frequently idiopathic, but may be associated with chronic inflammation, infection or neoplasia (DiBartola & Benson, 1989; Segev et al., 2012). Neoplastic conditions such as pancreatic endocrine tumour (O'Brien et al., 1987), ameloblastoma (Gardner & Dubielzig, 1993), plasmacytoma (Rowland et al., 1991), mammary tumour (Taniyama et al., 2000) and thymoma (Loewen et al., 2018) have previously been associated with amyloidosis in dogs. Because serum amyloid A (SAA) is directly involved in the pathogenesis of reactive amyloidosis (i.e., AA amyloidosis), it has been suggested that it may be a better marker for disease control and risk of AA amyloidosis than CRP in humans (Elhani et al., 2024). Results of studies comparing the diagnostic performance of canine CRP and SAA have been conflicting, with some suggesting that SAA may be an inferior marker compared to CRP, while others have shown that SAA has greater diagnostic potential for systemic inflammation (Christensen et al., 2014). In case 1, amyloidosis could have been secondary to the systemic inflammation, the undifferentiated SSS, or a combination of both. Although renal biopsies were not performed, renal amyloidosis was also suspected in this case given the severe, persistent proteinuria. Glomerular proteinuria that failed to resolve 2 weeks after surgery was also detected at admission in case 3. Besides amyloidosis, potential factors that could have contributed to the development of proteinuria in these cases include decreased renal blood flow, injury induced by products of tumour cells and deposition of antigen–antibody immune complexes (Lien & Lai, 2011). Cases 2 and 4 also showed mild to moderate proteinuria at presentation. However, glomerular disease was considered unlikely as proteinuria resolved after splenectomy. These findings should raise the concern of multi‐organic complications that could worsen the prognosis of dogs diagnosed with nodular splenic lesions with mixed components.

Case 1 had positive serologic titers for *Bartonella henselae*. An association between *Bartonella* spp. infection and nodular splenic masses has previously been described. A significantly higher prevalence of *Bartonella* spp. deoxyribonucleic acid (DNA) in splenic tissues from dogs with so‐called SFHN (29.7%) and HSA (26%) was found when compared with LNH (10%) and control spleens (0%). Also, the prevalence of *Bartonella* spp. DNA was significantly higher than other infectious agent (namely *Babesia* spp. or *Mycoplasma* spp.) DNA in these dogs (Varanat et al., 2011). One potential explanation is that chronic infection of macrophages by *Bartonella* spp. along with prevention of apoptosis leads to proliferation and formation of some nodular splenic lesions. Despite positive serologic titers, PCR testing on splenic tissue for *Bartonella* spp. was negative in our patient. The exact reason for the discrepancy between PCR and serologic testing is not fully understood. A false negative PCR result could have occurred because of the lack of sensitivity of PCR testing when performed without pre‐enrichment culture on Bartonella/alpha‐Proteobacteria growth medium (Duncan et al., 2007). Another potential explanation could be the small quantity of tissue placed in paraffin blocks from markedly enlarged spleen and the limited quantity of extracted DNA. Thus, it is unknown whether positive serologic testing reflected a truly active infection associated with splenic nodule development or indicated an old, clinically irrelevant infection.

Little information is available on the biological behaviour and outcomes of nodular splenic lesions after splenectomy. Unfortunately, most of the dogs in our series were lost to follow‐up, precluding long‐term investigation of the clinical behaviour of their splenic lesions. In addition, therapeutic guidelines following splenectomy are currently lacking. A recent study has provided evidence that canine splenic nodular lesions with mixed cell components and histological criteria of malignancy may behave aggressively, leading to metastasis and death (Sabattini et al., 2022). Another recent case series of 32 dogs confirmed the aggressive biological behaviour of SSS. Of these, 22 developed metastases with an incidence of 59% at 12 months, highlighting the potential benefit of adjuvant chemotherapy. However, the administration of chemotherapy did not reduce the risk of metastasis or affect survival in this cohort (Ferrari et al., 2024). Additionally, the use of toceranib had no effect on progression‐free survival or overall survival in dogs with HSA following splenectomy and doxorubicin‐based chemotherapy (Gardner et al., 2015). Therefore, further studies are needed to evaluate the efficacy of adjuvant therapeutic interventions and to guide treatment decisions in dogs with nodular splenic lesions. None of the dogs included in the present case series had received adjuvant therapy. In addition, diagnostic imaging was performed with thoracic radiography only and no dog had a CT scan. It is not possible to ensure that they did not have metastatic disease at inclusion or during follow‐up (Armbrust et al., 2012).

In conclusion, this study identified specific clinical and clinicopathological findings attributed to systemic inflammation in several cases of nodular splenic lesions composed of heterogeneous cell components, including undifferentiated SSS and splenic leiomyosarcoma. Based on the literature search, this is the first mid‐ to long‐term clinicopathological follow‐up described in dogs with nodular splenic lesions composed of heterogeneous cell components after splenectomy. The results of this report suggest that these lesions could be directly associated with both acute and chronic systemic inflammation leading to, for example, fever and polyclonal hypergammaglobulinaemia. Additionally, amyloidosis may be considered as a direct consequence of chronic inflammation. Thus, this report also highlights the need for an early detection and treatment of nodular splenic lesions with mixed components to prevent such complications, especially if the size of the nodule is associated with systemic inflammation.

### Conflict of interest

None of the authors of this article has a financial or personal relationship with other people or organisations that could inappropriately influence or bias the content of the paper.

## Author contributions

**K. Mourou:** Conceptualization (equal); data curation (equal); investigation (equal); project administration (lead); writing – original draft (lead); writing – review and editing (equal). **Y. Abou Monsef:** Investigation (supporting); resources (lead); writing – original draft (supporting); writing – review and editing (equal). **S. Belluco:** Conceptualization (supporting); investigation (supporting); resources (equal); supervision (equal); writing – review and editing (equal). **M. Penent:** Investigation (supporting); resources (equal); writing – review and editing (equal). **M. Delverdier:** Conceptualization (supporting); investigation (supporting); resources (equal); writing – review and editing (equal). **M. Hugonnard:** Conceptualization (supporting); investigation (supporting); supervision (equal); writing – review and editing (equal). **F. Granat:** Investigation (supporting); resources (equal); writing – review and editing (equal). **R. Lavoue:** Conceptualization (supporting); investigation (supporting); supervision (equal); writing – review and editing (equal). **M. Mantelli:** Conceptualization (equal); data curation (equal); investigation (equal); resources (equal); supervision (lead); validation (lead); writing – original draft (supporting); writing – review and editing (equal).

## Data Availability

The data that support the findings of this study are available from the corresponding author, [KM], upon reasonable request.

## References

[jsap13826-bib-0001] Armbrust, L.J. , Biller, D.S. , Bamford, A. , Chun, R. , Garrett, L.D. & Sanderson, M.W. (2012) Comparison of three‐view thoracic radiography and computed tomography for detection of pulmonary nodules in dogs with neoplasia. Journal of the American Veterinary Medical Association, 240, 1088–1094. 22515629 10.2460/javma.240.9.1088

[jsap13826-bib-0002] Backschat, P.S. , Nishiya, A.T. , Toyota, F.T. & Guerra, J.L. (2012) Estudo casuístico retrospectivo de neoformações primárias esplênicas [Retrospective case study of primary splenic neoformations]. Revista Científica de Medicina Veterinária, 10, 1–5.

[jsap13826-bib-0003] Christensen, M.B. , Langhorn, R. , Goddard, A. , Andreasen, E.B. , Moldal, E. , Tvarijonaviciute, A. et al. (2014) Comparison of serum amyloid A and C‐reactive protein as diagnostic markers of systemic inflammation in dogs. The Canadian Veterinary Journal, 55, 161–168. 24489396 PMC3894877

[jsap13826-bib-0004] Corvera, G. , Alegría‐Morán, R. , Cifuentes, F.F. & Torres, C.G. (2024) Pathological characterization and risk factors of splenic nodular lesions in dogs (*Canis lupus familiaris*). Animals (Basel), 14, 802. 38473187 10.3390/ani14050802 PMC10930829

[jsap13826-bib-0005] DiBartola, S.P. & Benson, M.D. (1989) The pathogenesis of reactive systemic amyloidosis. Journal of Veterinary Internal Medicine, 3, 31–41. 2647970 10.1111/j.1939-1676.1989.tb00326.x

[jsap13826-bib-0006] Duncan, A.W. , Maggi, R.G. & Breitschwerdt, E.B. (2007) A combined approach for the enhanced detection and isolation of *Bartonella* species in dog blood samples: pre‐enrichment liquid culture followed by PCR and subculture onto agar plates. Journal of Microbiological Methods, 69, 273–281. 17346836 10.1016/j.mimet.2007.01.010

[jsap13826-bib-0007] Eberle, N. , Von Babo, V. , Nolte, I. , Baumgärtner, W. & Betz, D. (2012) Splenic masses in dogs. Part 1: epidemiologic, clinical characteristics as well as histopathologic diagnosis in 249 cases (2000‐2011). Tierarztliche Praxis Ausgabe K Kleintiere Heimtiere, 40, 250–260. 22911256

[jsap13826-bib-0008] Elhani, I. , Jouret, M. , Malaise, O. , Nguyen, A.T. , Sarda, M.N. , Belot, A. et al. (2024) Performance of serum amyloid A and C reactive protein for disease control assessment in familial Mediterranean fever. The Journal of Allergy and Clinical Immunology. In Practice, S2213‐2198(24)01052‐3. 10.1016/j.jaip.2024.09.035 39389263

[jsap13826-bib-0040] Ettinger, S.J. , Feldman, E.C. & Cote, E. (2024) Textbook of veterinary internal medicine, 9th edition. St. Louis, MO, USA: Elsevier.

[jsap13826-bib-0009] Fernandez, S. , Lang, J.M. & Maritato, K.C. (2019) Evaluation of nodular splenic lesions in 370 small‐breed dogs (<15 kg). Journal of the American Animal Hospital Association, 55, 201–209. 31099604 10.5326/JAAHA-MS-6934

[jsap13826-bib-0010] Ferrari, R. , Marconato, L. , Boracchi, P. , Stefanello, D. , Godizzi, F. , Murgia, D. et al. (2024) Splenic stromal sarcomas in dogs: outcome and clinicopathological prognostic factors in 32 cases. Veterinary and Comparative Oncology, 22, 12–21. 37918913 10.1111/vco.12941

[jsap13826-bib-0011] Figueiredo, R.S. , Muramoto, C. , Fontes, T.N. , Meneses, I.D. , Cardoso, P.G. , Vieira Filho, C.H. et al. (2019) Lesions in 224 spleens of splenectomized dogs and evalution of alternative techniques for previous microscopic diagnosis. Pesquisa Veterinaria Brasileira, 39, 622–629.

[jsap13826-bib-0012] Gardner, D.G. & Dubielzig, R.R. (1993) The histopathological features of canine keratinizing ameloblastoma. Journal of Comparative Pathology, 109, 423–428. 7508956 10.1016/s0021-9975(08)80304-0

[jsap13826-bib-0013] Gardner, H.L. , London, C.A. , Portela, R.A. , Nguyen, S. , Rosenberg, M.P. , Klein, M.K. et al. (2015) Maintenance therapy with toceranib following doxorubicin‐based chemotherapy for canine splenic hemangiosarcoma. BMC Veterinary Research, 11, 131. 26062540 10.1186/s12917-015-0446-1 PMC4464614

[jsap13826-bib-0014] Klopfleisch, R. , Sperling, C. , Kershaw, O. & Gruber, A.D. (2011) Does the taking of biopsies affect the metastatic potential of tumours? A systematic review of reports on veterinary and human cases and animal models. Veterinary Journal, 190, 31–42. 10.1016/j.tvjl.2011.04.010 21723757

[jsap13826-bib-0015] Ko, J.‐S. , Kim, H. , Choi, Y. , Kim, J.W. , Park, C. & Do, S.‐H. (2013) Diagnostic approach to malignant fibrous histiocytomas of soft tissue in dogs: a case report. Veterinarni Medicina, 58, 621–627.

[jsap13826-bib-0016] Latifi, M. , Tuohy, J.L. , Coutermarsh‐Ott, S.L. , Klahn, S.L. , Leeper, H. & Dervisis, N. (2020) Clinical outcomes in dogs with localized splenic histiocytic sarcoma treated with splenectomy with or without adjuvant chemotherapy. Journal of Veterinary Internal Medicine, 34, 2645–2650. 32986268 10.1111/jvim.15910 PMC7694829

[jsap13826-bib-0017] Laurenson, M.P. , Hopper, K. , Herrera, M.A. & Johnson, E.G. (2010) Concurrent diseases and conditions in dogs with splenic vein thrombosis. Journal of Veterinary Internal Medicine, 24, 1298–1304. 20840302 10.1111/j.1939-1676.2010.0593.x

[jsap13826-bib-0018] Lee, M. , Park, J. , Choi, H. , Lee, H. & Jeong, S.M. (2018) Presurgical assessment of splenic tumors in dogs: a retrospective study of 57 cases (2012‐2017). Journal of Veterinary Science, 19, 827–834. 30173499 10.4142/jvs.2018.19.6.827 PMC6265589

[jsap13826-bib-0019] Lien, Y.‐H.H. & Lai, L.‐W. (2011) Pathogenesis, diagnosis and management of paraneoplastic glomerulonephritis. Nature Reviews. Nephrology, 7, 85–95. 21151207 10.1038/nrneph.2010.171 PMC3058941

[jsap13826-bib-0020] Loewen, J.M. , Cianciolo, R.E. , Zhang, L. , Yaeger, M. , Ward, J.L. , Smith, J.D. et al. (2018) Concurrent renal amyloidosis and thymoma resulting in a fatal ventricular thrombus in a dog. Journal of Veterinary Internal Medicine, 32, 1160–1165. 29485186 10.1111/jvim.15062 PMC5980280

[jsap13826-bib-0042] Maxie, M.G. (2015) Jubb, Kennedy & Palmer's pathology of domestic animals: volume 1–3, 6th edition. St. Louis, MO, USA: Elsevier.

[jsap13826-bib-0021] Moore, A.S. , Frimberger, A.E. , Sullivan, N. & Moore, P.F. (2012) Histologic and immunohistochemical review of splenic fibrohistiocytic nodules in dogs. Journal of Veterinary Internal Medicine, 26, 1164–1168. 22882592 10.1111/j.1939-1676.2012.00986.x

[jsap13826-bib-0022] Morris, J.S. , McInnes, E.F. , Bostock, D.E. , Hoather, T.M. & Dobson, J.M. (2002) Immunohistochemical and histopathologic features of 14 malignant fibrous histiocytomas from flat‐coated retrievers. Veterinary Pathology, 39, 473–479. 12126150 10.1354/vp.39-4-473

[jsap13826-bib-0023] O'Brien, T.D. , Hayden, D.W. , O'Leary, T.P. , Caywood, D.D. & Johnson, K.H. (1987) Canine pancreatic endocrine tumors: immunohistochemical analysis of hormone content and amyloid. Veterinary Pathology, 24, 308–314. 2887054 10.1177/030098588702400404

[jsap13826-bib-0024] O'Brien, D. , Moore, P.F. , Vernau, W. , Peauroi, J.R. , Rebhun, R.B. , Rodriguez, C.O., Jr. et al. (2013) Clinical characteristics and outcome in dogs with splenic marginal zone lymphoma. Journal of Veterinary Internal Medicine, 27, 949–954. 23734665 10.1111/jvim.12116 PMC5012422

[jsap13826-bib-0025] O'Byrne, K. & Hosgood, G. (2019) Splenic mass diagnosis in dogs undergoing splenectomy according to breed size. The Veterinary Record, 184, 620–625. 31040215 10.1136/vr.104983

[jsap13826-bib-0043] Raskin, R.E. , Meyer, D.J. & Boes, K.M. (2022) Canine and Feline cytology, 4th edition. St. Louis, MO, USA: Elsevier.

[jsap13826-bib-0026] Rowland, P.H. , Valentine, B.A. , Stebbins, K.E. & Smith, C.A. (1991) Cutaneous plasmacytomas with amyloid in six dogs. Veterinary Pathology, 28, 125–130. 1712141 10.1177/030098589102800204

[jsap13826-bib-0027] Sabattini, S. , Lopparelli, R.M. , Rigillo, A. , Giantin, M. , Renzi, A. , Matteo, C. et al. (2018) Canine splenic nodular lymphoid lesions: immunophenotyping, proliferative activity, and clonality assessment. Veterinary Pathology, 55, 645–653. 29807508 10.1177/0300985818777035

[jsap13826-bib-0028] Sabattini, S. , Rigillo, A. , Foiani, G. , Marconato, L. , Vascellari, M. , Greco, A. et al. (2022) Clinicopathologic features and biologic behavior of canine splenic nodules with stromal, histiocytic and lymphoid components. Frontiers in Veterinary Science, 9, 962685. 36032303 10.3389/fvets.2022.962685 PMC9411940

[jsap13826-bib-0029] Segev, G. , Cowgill, L.D. , Jessen, S. , Berkowitz, A. , Mohr, C.F. & Aroch, I. (2012) Renal amyloidosis in dogs: a retrospective study of 91 cases with comparison of the disease between Shar‐Pei and non‐Shar‐Pei dogs. Journal of Veterinary Internal Medicine, 26, 259–268. 22268374 10.1111/j.1939-1676.2011.00878.x

[jsap13826-bib-0030] Sherwood, J.M. , Haynes, A.M. , Klocke, E. , Higginbotham, M.L. , Thomson, E.M. , Weng, H.Y. et al. (2016) Occurrence and clinicopathologic features of splenic neoplasia based on body weight: 325 dogs (2003–2013). Journal of the American Animal Hospital Association, 52, 220–226. 27259024 10.5326/JAAHA-MS-6346

[jsap13826-bib-0031] Spangler, W.L. & Kass, P.H. (1998) Pathologic and prognostic characteristics of splenomegaly in dogs due to fibrohistiocytic nodules: 98 cases. Veterinary Pathology, 35, 488–498. 9823590 10.1177/030098589803500603

[jsap13826-bib-0032] Spröhnle‐Barrera, C.H. , McGhie, J. , Allavena, R.E. , Owen, H.C. , Palmieri, C. & Barnes, T.S. (2022) Epidemiology and survival of dogs diagnosed with splenic lymphoid hyperplasia, complex hyperplasia, stromal sarcoma and histiocytic sarcoma. Animals, 8, 960. 10.3390/ani12080960 PMC9028997 35454207

[jsap13826-bib-0033] Taniyama, H. , Kitamura, A. , Kagawa, Y. , Hirayama, K. , Yoshino, T. & Kamiya, S. (2000) Localized amyloidosis in canine mammary tumors. Veterinary Pathology, 37, 104–107. 10643991 10.1354/vp.37-1-104

[jsap13826-bib-0034] Tappin, S.W. , Taylor, S.S. , Tasker, S. , Dodkin, S.J. , Papasouliotis, K. & Murphy, K.F. (2011) Serum protein electrophoresis in 147 dogs. The Veterinary Record, 168, 456. 21493443 10.1136/vr.d88

[jsap13826-bib-0035] Tecilla, M. , Gambini, M. , Forlani, A. , Caniatti, M. , Ghisleni, G. & Roccabianca, P. (2019) Evaluation of cytological diagnostic accuracy for canine splenic neoplasms: an investigation in 78 cases using STARD guidelines. PLoS One, 14, e0224945. 31697755 10.1371/journal.pone.0224945 PMC6837434

[jsap13826-bib-0041] Vail, D.M. , Thamm, D.H. & Liptak, J.M. (2019) Withrow & MacEwen's small animal clinical oncology, 6th edition. St. Louis, MO, USA: Elsevier.

[jsap13826-bib-0036] Van den Broek Campanelli, T. , Alves, D.L. , Lima, S.R. , Braga, G. & Dall'Olio, A.J. (2021) Estudo Retrospectivo de exames histopatologicos esplenic na rotina laboratorial do Hospital Escola Veterinario UNIFAJ de 2015 A 2020 [Retrospective study of histopathologic splenic exams in the routine laboratory of the Veterinary School Hospital UNIFAJ from 2015 to 2020]. Veterinaria e Zootecnia, 28, 1–7.

[jsap13826-bib-0037] Varanat, M. , Maggi, R.G. , Linder, K.E. & Breitschwerdt, E.B. (2011) Molecular prevalence of *Bartonella*, *Babesia*, and hemotropic *Mycoplasma* sp. in dogs with splenic disease. Journal of Veterinary Internal Medicine, 25, 1284–1291. 22092618 10.1111/j.1939-1676.2011.00811.x

[jsap13826-bib-0038] Vilar Saavedra, P. , Lara García, A. , Zaldívar López, S. & Couto, G. (2011) Hemostatic abnormalities in dogs with carcinoma: a thromboelastographic characterization of hypercoagulability. Veterinary Journal, 190, 78–83. 10.1016/j.tvjl.2011.02.025 21454111

[jsap13826-bib-0044] Wennogle, S.A. , Olver, C.S. & Shropshire, S.B. (2021) Coagulation status, fibrinolysis, and platelet dynamics in dogs with chronic inflammatory enteropathy. Journal of Veterinary Internal Medicine, 35, 892–901. 33665845 10.1111/jvim.16092 PMC7995439

[jsap13826-bib-0039] Wittenberns, B.M. , Thamm, D.H. , Palmer, E.P. & Regan, D.P. (2021) Canine non‐angiogenic, non‐myogenic splenic stromal sarcoma: a retrospective clinicopathological analysis and investigation of podoplanin as a marker of tumour histogenesis. Journal of Comparative Pathology, 188, 1–12. 34686271 10.1016/j.jcpa.2021.07.006 PMC8542103

